# A Replication Study for the Association of rs726252 in PAPPA2 with Developmental Dysplasia of the Hip in Chinese Han Population

**DOI:** 10.1155/2014/979520

**Published:** 2014-02-03

**Authors:** Dongquan Shi, Wei Sun, Xingquan Xu, Zheng Hao, Jin Dai, Zhihong Xu, Dongyang Chen, Huajian Teng, Qing Jiang

**Affiliations:** ^1^The Center of Diagnosis and Treatment for Joint Disease, Drum Tower Hospital Affiliated to Medical School of Nanjing University, Nanjing, Jiangsu 210008, China; ^2^Laboratory for Bone and Joint Diseases, Model Animal Research Center, Nanjing University, Nanjing, Jiangsu 210061, China; ^3^The Center of Diagnosis and Treatment for Joint Disease, Nanjing Drum Tower Hospital, Clinical College of Nanjing Medical University, Nanjing, Jiangsu 210008, China; ^4^Center of Diagnosis and Treatment for Developmental Dysplasia of Hip, Kang'ai Hospital, Nanjing, Jiangsu 210008, China

## Abstract

Developmental dysplasia of the hip (DDH) is a common developmental hip disorder, which ranges from mild acetabulum malformation to irreducible hip dislocation. A previous study suggested a significant association of pregnancy-associated plasma protein-A2 (PAPPA2) with DDH susceptibility in Chinese Han population. But with the consideration of the sample size, the association was still debatable. To confirm the association of the reported single nucleotide polymorphism (SNP) in PAPPA2, rs726252 with DDH, we conducted a case-control study in a larger number of subjects. We genotyped rs726252 in 697 DDH subjects and 707 control subjects by TaqMan assay. The association between this SNP and DDH was evaluated statistically. No significant difference was found in any comparison of genotype distribution nor allele frequency between cases and controls. Our replication study indicated that the association between rs726252 and DDH in Chinese Han population was debatable. The association between PAPPA2 and DDH should be evaluated by additional studies.

## 1. Introduction

Developmental dysplasia of the hip (DDH) is a common developmental hip disorder, which ranges from mild acetabulum malformation to irreducible hip dislocation [1, 2]. DDH is a main cause of childhood disability and early onset hip osteoarthritis [3–5], and an epidemiologic study showed that DDH underlies up to 9% and 29% of all primary hip arthroplasty and those in people younger than 60 years, respectively [6]. DDH is affected by multiple factors including environmental and genetic factors. Family history of DDH, female gender, breech positioning at delivery, and large birth weight are risk factors of DDH [7]. In previous studies, we had detected associations between DDH and single nucleotide polymorphisms (SNPs) in GDF5, TBX4, and ASPN by case-control studies in Chinese Han population [8–10], and the association between DDH and GDF5 was also found in Caucasians [11].

Recently, a genome-wide linkage scan was conducted in a four-generation Chinese family of 25 members which includes 5 DDH patients. An association study based on the linkage scan was executed [12], and a SNP in pregnancy-associated plasma protein-A2 gene (PAPPA2), rs726252, was found to be associated with DDH. PAPPA2 encodes a novel metalloproteinase pregnancy-associated plasma protein-A2 which may play roles in fetal development [13–16]. In the comparison between 310 DDH patients and 487 control subjects, they detected significant differences of both genotype distribution and allele frequency of rs726252 (*P* = 0.0014 and 0.0013, resp.). Significant differences of genotype distribution and allele frequency still existed after stratification by gender (female *P* = 0.019 and 0.0079, male *P* = 0.0065 and 0.0035). The study suggested a significant association of PAPPA2 with DDH susceptibility in Chinese Han population. But with the consideration of the sample size, we thought the association between rs726252 and DDH was still debatable. To confirm the association of this SNP with DDH, we conducted a case-control study in a larger number of subjects.

## 2. Materials and Methods 

### 2.1. Subjects

A total of 1404 subjects including 697 DDH patients and 707 healthy control subjects were enrolled in the study. The patients (594 females and 103 males) were consecutively recruited from the Center of Diagnosis and Treatment for DDH, Kang'ai Hospital, Nanjing, China, and the healthy control subjects (299 females and 408 males) were enrolled from the Physical Examination Center, Drum Tower Hospital Affiliated to Medical School of Nanjing University, China. The patients were diagnosed as unilateral or bilateral DDH by expert medical examination with radiographic evidence. The criteria of defining the severity of DDH were as previously described [8]. Patients with systemic syndrome were excluded from this study. The control subjects were identified by physical examination and detailed history, and they had no symptoms or histories of DDH. All subjects in the study were unrelated Han Chinese living in or around Nanjing, Southern China. No subjects dropped out during the process of the study. The study was approved by the ethical committee of Drum Tower Hospital Affiliated to Medical School of Nanjing University, and informed consent was obtained from patients and controls.

### 2.2. Genotyping

DNA was obtained from all the subjects from peripheral blood using the NucleoSpin Blood QuickPure Kit (Macherey-Nagel, German) according to the manufacturer's instructions. The SNP rs726252 was genotyped using TaqMan assay on an ABI 7500 real-time polymerase chain reaction (PCR) instrument (Applied Biosystems 7500, ABI, Foster City, CA, USA). Genotyping was performed by laboratory personnel blinded to case status, and two authors independently reviewed the genotyping results, data entry, and statistical analyses.

### 2.3. Statistics

In this case-control study, we used standard chi-square analysis to compare the PAPPA2 genotype and allele distributions. Hardy-Weinberg equilibrium was also performed by chi-square test. The associations between PAPPA2 variants and DDH risk were evaluated by computing the odds ratios (ORs) and 95% confidence intervals (CIs) stratified by gender and severity. Meta-analysis of two studies was also conducted. All these statistical analyses were performed with SAS 9.1.3 software (SAS Institute, Cary, NC). The results were shown in Tables 1, 2, 3, and 4.

## 3. Result

**Figure 1:**
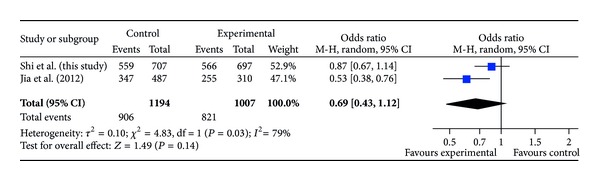
ORs of risk allele frequency (RAF) in case-control study.

**Figure 2:**
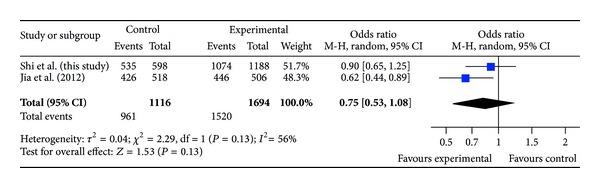
ORs of risk allele frequency (RAF) in case-control study for female subjects.

**Figure 3:**
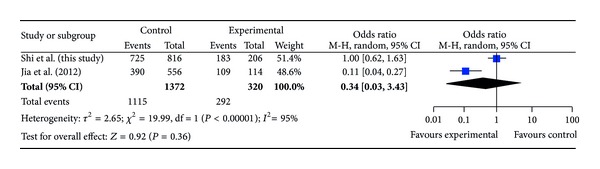
ORs of risk allele frequency (RAF) in case-control study for male subjects.

The ages of DDH patients and controls (mean ± SD) were 23.2 ± 11.8 months (range, 2 to 83 months) and 56.2 ± 12.7 years (range, 34 to 76 years), respectively. The distributions of the alleles and genotypes for rs726252 are presented in Table 1. Distributions of genotypes in the DDH and control groups were conformed to Hardy-Weinberg equilibrium (*P* = 0.75 and 0.36, resp.). The minor allele frequency of rs726252 of controls in our study was 0.11, and it was closer to that reported in HapMap for Chinese Han populations (0.08) than the previous study [12]. We compared the genotype frequencies and allele frequencies between the patients and the controls; no significant difference was observed (*P* = 0.59 and 0.36, resp.). No significant difference was found in any comparison after stratification by gender and severity of DDH (Tables 2 and 3). We also compared allele frequency and genotype distribution in subjects between our study and the previous one. We found a significant difference of genotype distribution between our cases and Jia's cases (*P* = 0.011) and significant differences of allele frequency and genotype between our control subjects and Jia's control subjects (*P* = 0.0001 and 0.0002, resp.) (Table 4). In meta-analysis for the two studies, ORs of risk allele frequency (RAF) in case-control study are shown in Figure 1, ORs of RAF when stratified by gender are shown in Figures 2 and 3. Both in Figures 1 and 2, the allelic ORs are less than 1; however, in Figure 3, we found that OR in our study is different from Jia's.

## 4. Discussion

In this study, a total of 1404 individuals were analyzed. For this SNP (rs726252), we did not detect any significant difference in genotype distribution or allele frequency between cases and controls, even after stratification by gender and severity of DDH. Our study indicated that rs726252 in PAPPA2 had no association with susceptibility to DDH in the Chinese Han population. Thus, our results do not support the previous results [12].

By comparison of allele frequency and genotype distribution in subjects between the two studies, there was no significant difference in comparison of allele frequency between our cases and Jia's cases, but significant difference of genotype distribution was found between the case subjects in two studies. Significant differences were found in comparison of allele frequency and genotype distribution between control subjects in the two studies (*P* = 0.0001 and 0.0002, resp.). We checked this SNP in HapMap database; the minor allele frequency was 0.08, and the minor allele frequency of control subjects of the two studies was 0.11 and 0.16. The minor allele frequency of this SNP of control subjects in our study was closer to that in Chinese population of HapMap database. The sample size in our study is twice larger than theirs, and it makes our data more reliable. So we inferred that there might be a sampling error in the previous study. Although the genetic difference between Southern China and North China is very small [17], it is also a potential influence factor for the different results.

There are also several limitations in the current study. First, due to low prevalence of DDH in males, the number of male subjects in our study was relatively limited. The sample size of hip instability and subluxation was also limited. We are continuously collecting both male and female subjects. Second, we only tested one SNP, rs726252 in the present study. We can only deny the association between this SNP and DDH. The association between this gene and DDH is still suspicious.

In conclusion, our replication study indicated that there was no association between rs726252 and DDH in Chinese Han population. The association between PAPPA2 and DDH was suspicious and it still needed further evaluation.

## Figures and Tables

**Table 1:** Genotype and allele frequencies of C/T transition in single nucleotide polymorphism (SNP) (rs726252) of the PAPPA2 gene in Han Chinese population.

Subjects	Number	Allele		Genotype			Hardy-Weinberg equilibrium *P* value
		T (%)	C (%)	TT (%)	TC (%)	CC (%)	
All cases	697	1257 (90.2)	137 (9.8)	566 (81.2)	125 (17.9)	6 (0.9)	0.754
All controls	707	1260 (89.1)	154 (10.9)	559 (79.1)	142 (20.1)	6 (0.8)	0.355
Female cases	594	1074 (90.4)	114 (9.6)	485 (81.6)	104 (17.5)	5 (0.8)	0.824
Female controls	299	535 (89.5)	63 (10.5)	237 (79.3)	61 (20.4)	1 (0.3)	0.155
Male cases	103	183 (88.8)	23 (11.2)	81 (78.6)	21 (20.4)	1 (1.0)	0.778
Male controls	408	725 (88.8)	93 (11.2)	322 (78.9)	81 (19.9)	5 (1.2)	0.970

**Table 2:** Association of rs726252 in PAPPA2 with developmental dysplasia of the hip when stratified by gender.

Groups compared	TT versus other combined			CC versus other combined			T allele versus C allele			All genotypes
	*P* value	OR	95% CI	*P* value	OR	95% CI	*P* value	OR	95% CI	*P* value
All patients (*n* = 697) versus all controls (*n* = 707)	0.315	0.874	0.672 to 1.137	0.980	1.015	0.326 to 3.161	0.355	0.892	0.699 to 1.137	0.590

Female patients (*n* = 594) versus female controls (*n* = 299)	0.393	0.859	0.606 to 1.217	0.381	2.530	0.294 to 21.751	0.531	0.901	0.652 to 1.247	0.406

Male patients (*n* = 103) versus male controls (*n* = 408)	0.950	1.017	0.600 to 1.724	0.830	0.790	0.091 to 6.838	0.996	1.001	0.616 to 1.627	0.971

**Table 3:** Association of rs726252 in PAPPA2 with developmental dysplasia of the hip when stratified by severity.

Groups compared	TT versus other combined			CC versus other combined			T allele versus C allele			All genotypes
	*P* value	OR	95% CI	*P* value	OR	95% CI	*P* value	OR	95% CI	*P* value
Patients with hip instability (*n* = 36) versus all controls (*n* = 707)	0.538	0.755	0.309 to 1.849	0.243	3.338	0.391 to 28.488	0.756	0.881	0.397 to 1.956	0.351

Patients with hip subluxation (*n* = 95) versus all controls (*n* = 707)	0.491	0.823	0.473 to 1.434	0.841	1.243	0.148 to 10.438	0.553	0.856	0.512 to 1.431	0.747

Patients with hip dislocation (*n* = 566) versus all controls (*n* = 707)	0.413	0.891	0.675 to 1.175	0.776	0.832	0.234 to 2.961	0.414	0.898	0.695 to 1.161	0.708

**Table 4:** Genotype and allele frequencies of rs726252 of the PAPPA2 gene in two Chinese groups.

Subjects	Number	Allele			Genotype			
		T (%)	C (%)	*P* value	TT (%)	TC (%)	CC (%)	*P* value
Cases	697	1257 (90.2)	137 (9.8)	0.651	566 (81.2)	125 (17.9)	6 (0.9)	0.011
Cases*	310	555 (89.5)	65 (10.5)		255 (82.2)	45 (14.5)	10 (3.3)	
Controls	707	1260 (89.1)	154 (10.9)	0.0001	559 (79.1)	142 (20.1)	6 (0.8)	0.0002
Controls*	299	816 (83.8)	158 (16.2)		347 (71.3)	122 (25.1)	18 (3.7)	

*The allele and genotype frequency of rs725262 in Jia's study.
